# Supplementary material for: Background-free 12C(α, γ) angular distribution measurements with a time projection chamber operating in Gamma beams
Source: Commun Phys. 2026 Jan 6;9(1):27. doi: 10.1038/s42005-025-02458-7 (PMC12823443; doi:10.1038/s42005-025-02458-7)
Supplement: Supplementary file 2 — Supplementary material [file 42005_2025_2458_MOESM2_ESM.pdf]

Supplementary Information for Background-free $^{12}C(\alpha, \gamma)$ Angular Distribution Measurements with a Time Projection Chamber Operating in Gamma Beams

Kristian C. Z. Haverson[1,*], Robin Smith[1,2], Moshe Gai[2], Deran K. Schweitzer[2], Sarah R. Stern[2], and Sean W. Finch[3]

[1]School of Engineering & Built Environment, Sheffield Hallam University, Sheffield, S1 1WB, UK

[2]Laboratory for Nuclear Science at Avery Point, University of Connecticut, Groton, CT 06340-6097, USA

[3]Department of Physics and Triangle Universities Nuclear Laboratory, Duke University, Durham, NC 27708-0308, USA

[*]Corresponding author: kristianzajdek@gmail.com

November 2025

1 Supplementary Figure 1

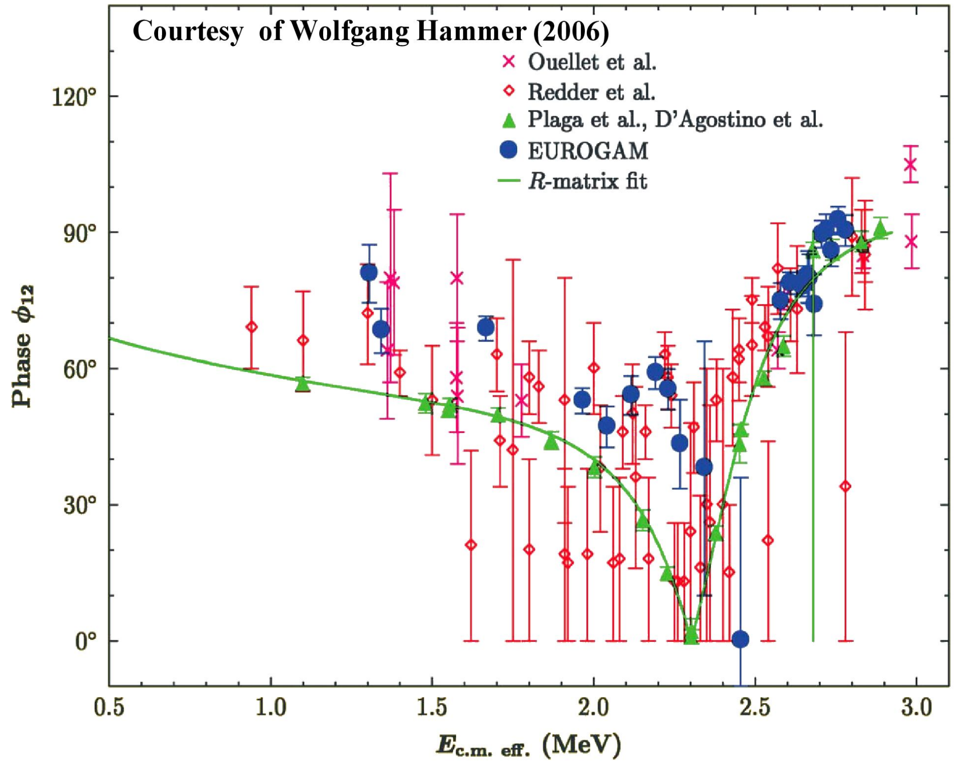

Figure 1: **Comparison of measured ϕ_{12} from the world data with the theoretical prediction from elastic scattering data of Plaga *et al.* [1] and D'Agostino *et al.* [2].** This figure was prepared by Wolfgang Hammer and is reproduced here with permission.

2 Supplementary Figure 2

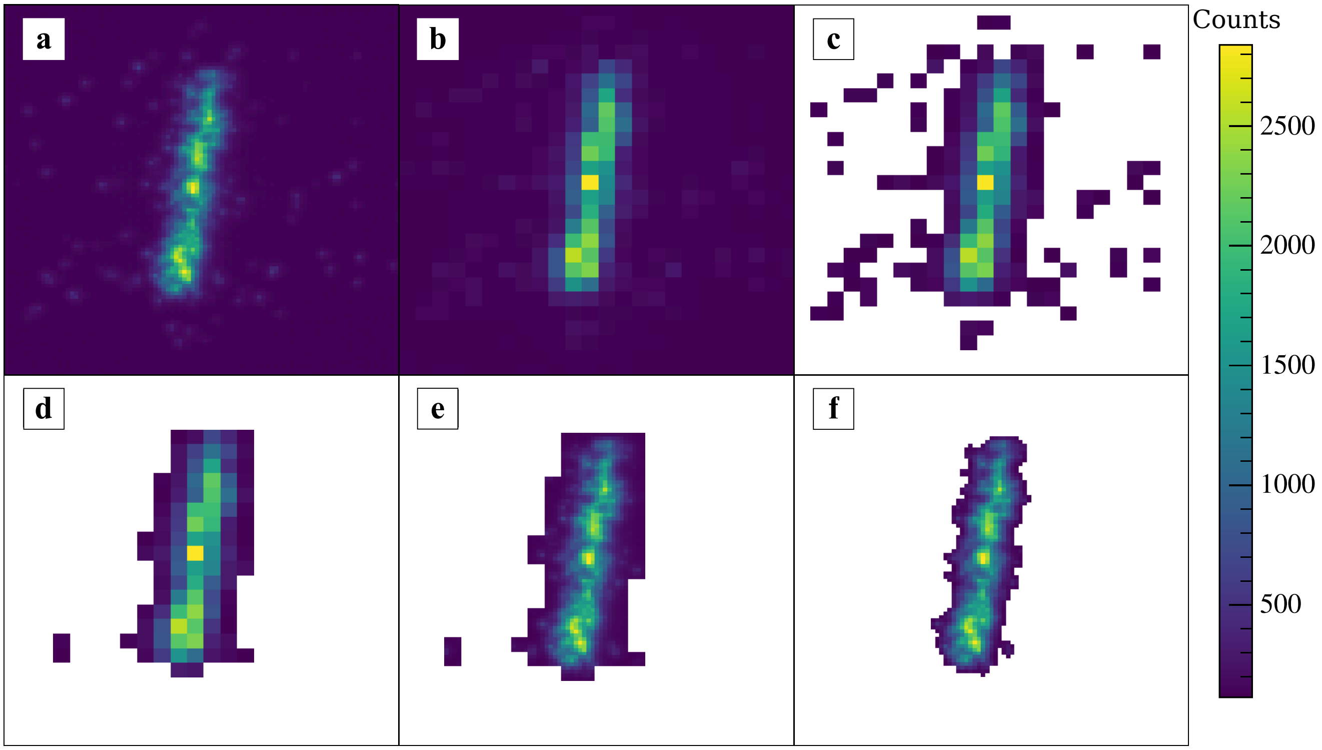

Figure 2: **Image processing steps for the photo-dissociation and cosmic events.** A series of CCD image processing steps used to isolate the particle tracks. Numbers in the colour bar correspond to the full resolution image and should be scaled by the 4×4 degree of pixelation for the lower resolution panels. (a) A flat-field-corrected $^{16}O(\gamma, \alpha)$ image. (b) The same image pixelated horizontally and vertically by a factor of 4. (c) The image after a background threshold was applied. (d) The image after zeroing pixels with fewer than 5 non-zero neighbouring pixels. (e) Restoration of the original image resolution. (f) All pixels outside of the largest cluster are zeroed, and a final finer neighbour scan is applied.

3 Supplementary Figure 3

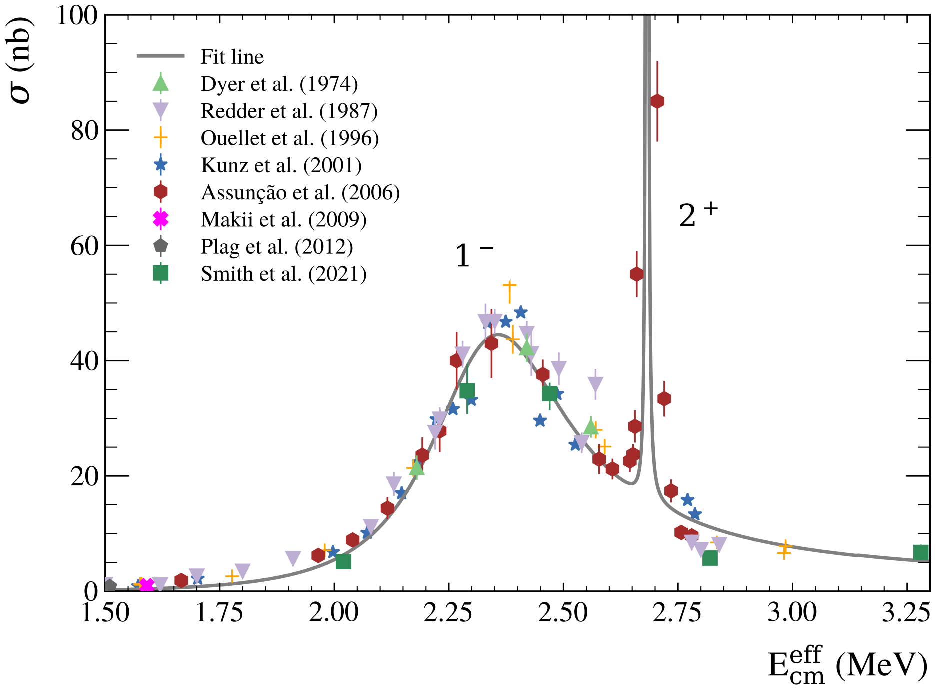

Figure 3: **Phenomenological fit to the total cross section world data.** The overall shape of the cross section was modelled as a sum of a broad 1^- and a narrow 2^+ resonance. The aim of this was not to perform a full R-matrix fit, but rather to parametrise the shape of the observed cross section in a simple way.

Supplementary References

[1] R. Plaga, *et al.*, The scattering of alpha particles from ^{12}C and the ^{12}C$(\alpha,\gamma)^{16}$O stellar reaction rate. Nuclear Physics A **465(2)**, 291 (1987).

[2] M. Bruno, *et al.*, Experimental study of the α-^{12}C elastic scattering. R-matrix analysis of the phase shifts and 16O levels. Il Nuovo Cimento A **27(1)**, 1–26 (1975).
