# Supplementary material for: Background-free 12C(α, γ) angular distribution measurements with a time projection chamber operating in Gamma beams
Source: Commun Phys. 2026 Jan 6;9(1):27. doi: 10.1038/s42005-025-02458-7 (PMC12823443; doi:10.1038/s42005-025-02458-7)
Supplement: Supplementary file 3 — Description of Additional Supplementary Files [file 42005_2025_2458_MOESM3_ESM.docx]

**Description of Additional Supplementary Files**

File name- Supplementary Data

File description-
